# Regulation of Root Development and Architecture by Strigolactones under Optimal and Nutrient Deficiency Conditions

**DOI:** 10.3390/ijms19071887

**Published:** 2018-06-27

**Authors:** Marek Marzec, Michael Melzer

**Affiliations:** 1 Department of Genetics, Faculty of Biology and Environmental Protection, University of Silesia, 40-032 Katowice, Poland; 2 Department of Physiology and Cell Biology, Leibniz Institute of Plant Genetics and Crop Plant Research (IPK), D-06466 Gatersleben, Germany; melzer@ipk-gatersleben.de

**Keywords:** nutrient stress, root development, strigolactones (SLs)

## Abstract

Strigolactones (SLs) constitute a group of plant hormones which are involved in multiple aspects of plant growth and development. Beside their role in shoot and root development and plant architecture in general, SLs are also involved in plant responses to nutrient deficiency by promoting interactions with symbiotic organisms and via promotion of root elongation. Recent observations on the cross talk between SLs and other hormones demonstrate that the inhibition of adventitious root formation by ethylene is independent of SLs. Additionally, it was shown that root exposure to SLs leads to the accumulation of secondary metabolites, such as flavonols or antioxidants. These data suggest pleiotropic effects of SLs, that influence root development. The discovery that the commonly used synthetic SL analogue racGR24 might also mimic the function of other plant growth regulators, such as karrikins, has led us to consider the previously published publications under the new aspects. This review summarizes present knowledge about the function of SLs in shaping root systems under optimal and nutrient deficiency conditions. Results which appear inconsistent with the various aspects of root development are singled out.

## 1. Introduction

Strigolactones (SLs) are a class of plant hormones first identified as such in 2008 as shoot branching factors [1,2]. Before this, SLs had already been known to stimulate germination of parasitic plant seeds [3] and to promote the interaction with arbuscular mycorrhizal fungi (AMF) [4]. It has become clear that SLs not only play a role in the shaping of shoot architecture in different species [5], but also in root development, as well as in promoting nodulation in *Pisum sativum* L. [6], and also in leaf senescence [7]. On top of this, SLs also appear to be integral parts of the plant responses to biotic and abiotic stresses. Under both P or N deficiency conditions, synthesis of SLs is increased, and larger amounts of this hormone are secreted into the soil, probably to promote the symbiotic relations with AMF and bacteria. The elevated levels of SLs also influence the plant phenotype by suppressing shoot growth and stimulating root development, thus adapting the plant to starvation conditions (for review see [8,9,10]). The contribution of SLs in plant adaptation to the other stresses, such as drought was postulated. According to some recent studies SL mutants of *Arabidopsis thaliana* L. are more sensitive to drought stress in comparison to the wild-type plants [11,12]. Additional studies on *Lotus japonicas* L. [13] and *Solanum lycopersicum* L. [14,15] confirmed that SLs together with abscisic acid play a role in plant adaptation to the limited water conditions. Additionally, in response to drought, tomato plants show decreased SL biosynthesis in the roots but increased biosynthesis in the shoots [15]. However, the role of SLs in plant response to drought remains unclear, and recent reports indicated that some aspect drought resistance of SL mutants, might be karrikin (KAR)-dependent [16]. Nevertheless, results like these feed the impression that SLs may well present a broad-spectrum class of phytohormones. Some time ago, based on an in silico analysis of the genes involved in SL biosynthesis in *A. thaliana* and *Oryza sativa* L. it has been postulated that SLs may also participate in plant responses to wounding, cold stress or flooding and the cross-talk between SLs and other class of hormones was predicted [17]. Recently, the experimental evidences has confirmed the role of SLs in plant resistance to bacterial and fungal pathogens [18].

SLs are biosynthesized in roots and transported to the shoot via SL transporter PLEIOTROPIC DRUG RESISTANCE 1 (PDR1) [19], but they might be also produced in the above-ground parts of plants [20]. SLs are produced in plastids and biosynthesis starts with the conversion of all-*trans*-β-carotene through a carotenoid isomerase and two carotenoid cleavage dioxygenases [21]. Carlactone, the conversion product, is exported into the cytoplasm where it is converted into 5-deoxystrigol or orobanchol [22], the main precursors of more than 20 naturally occurring SLs [23]. All known SLs share a similar structure, composed of a tricyclic lactone (ABC ring) connected to a butenolide group (D ring) by an enol-ether bond. SLs are divided into two groups based on the stereochemical differences at the junctions between B and C rings: the orobanchol group with an α-oriented C ring and the strigol group with a β-oriented C ring [24]. Further details on SL biosynthesis have been described previously [25,26].

In recent years, SL perception and signalling including the complete SL signal transduction pathway have been unravelled [27]. SLs are recognized by receptors from the α/β hydrolase family. Hydrolysis of the SL molecules alters the conformation of the receptor itself [28,29,30]. This allows the interaction of the SKP1-CULLIN-F-BOX complex (SCF) with the SL receptor [31] which subsequently is degraded in the proteasome [30,32]. Studies on SLs are confounded by the fact that not all components of the SL signalling pathway are specific for this group of hormones. For example, the F-box protein from the SCF complex—MAX2—is also involved in the signalling pathway of KARs and some aspects of the *max2* phenotype are more KAR- than SL-dependent [26,33]. Even racGR24, the synthetic analogue of SLs frequently used in SL studies, is a mixture of two 5-deoxystrigol (5DS) configured enantiomers: GR24^5DS^ and GR24^ent-5DS^, that may differently influence both SL and KAR signalling pathways [34]. This means that studies using *max2* mutants and/or racGR24 have to be critically interpreted, since the observed phenotype might be caused by stimulation or disorder in the KAR signalling pathway. The prevailing view is that application of SL biosynthesis mutants or mutants with disorders in SL receptor should become the gold standard in SL studies [26]. The present review summarizes the studies on SLs in root development and highlights those results that might be explained by using racGR24 and/or *max2* mutants instead of plants with a proven mutation in SL receptor D14 (DWARF14).

## 2. Role of SLs in Root System Development, under Optimal Growth Conditions

Studies on *A. thaliana* provided the first evidences that SLs may be involved in the control of root system architecture. Under optimal growth conditions mutants of SL biosynthesis (*max3-11*, *max4-1*) and SL signalling (*max2-1*) developed more lateral roots (LRs) compared to wild-type plants [35]. Treatment with 2.5 and 5 µM of racGR24 reduced LRs density in both wild-type plants and mutants of SL biosynthesis, but did not affect LR density in SL signalling mutants (Table 1). High concentrations of racGR24 (10 µM) decreased the number of LR in *max2* signalling mutant in an MAX2-independet manner [35,36]. It turned out that though racGR24 reduced the number of LRs, and had no effect on already formed ones, indicating that SLs are only involved in the initiation of LRs [37]. In barley (*Hordeum vulgare* L.) the SL signalling mutant *hvd14.d* also displayed a higher density of LRs but showed no differences in LR length compared to the wild-type [38]. Treatment with racGR24 decreased the number of LRs in wild-type barley plants, but not in the *hvd14.d* mutant [38]. Similar results were observed in *Lotus japonicus* L. where lines silenced for one of the SL biosynthesis genes (*LjCCD7*) showed more LRs [39], and in *Medicago truncatula* Gaertner (Gaertn.) where racGR24 treatment of wild-type plants resulted in lower LR density [40]. In rice, however, SL biosynthesis (*d10*) or SL signalling (*d14*) mutants possessed similar numbers of LRs as wild-type [41].

While it, thus, appeared that racGR24 had an overall inhibitory effect of LR formation in all wild-type plants tested, analysis of *d14* mutants in rice and barley, however, indicated that a higher number of LRs was only present in the barley mutant [38,41]. These contradictory results may be caused by external factors: barley plants were grown in vermiculite without additional supplementation [38], whereas rice was grown in hydroponics in ½ Murashige and Skoog media [41]. An alternative explanation may be the different root developmental programs between barley and rice. Whereas young barley seedlings have long multiple and branched seminal roots (SRs) and only a few short crown roots (CRs) [38], young rice seedlings display a single short SR but multiple CRs [41].

Reports on SL effect on root length are inconsistent (Table 1). While racGR24 did not influence root length in *Solanum lycopersicon* L., it did promote cell elongation during auxin (IAA) treatment, thus reversing the suppressive effect of IAA [42]. In *A. thaliana* grown under optimal conditions primary root (PR) length of SL mutants and wild-type plants were similar, but a positive effect of racGR24 treatment was present under carbohydrate starvation [36]. In rice the length of SRs of SLs mutants was comparable to that of wild-type [41], but in barley SRs of the *hvd14.d* mutant were distinctly shorter than those of the wild-type [38]. In both, rice and barley, treatment with racGR24 promoted elongation of SRs in wild-type plants [38,43]. In contrast to this RNAi transgenic lines for *LjCCD7* of *L. japonicus* had longer PRs, suggesting that in this species SLs suppress PR growth [39]. According to these results the effect of SLs on root elongation seems to be species specific, but the effects of SLs on shoot architecture are identical in both mono- and dicots. Obviously, root phenotypes of SL-mutants in different species have to be examined under highly controlled conditions for a proper evaluation of SL effects.

The role of SLs in CR development has so far only been investigated in rice (Table 1). Although wild-type and SL mutants displayed similar numbers of CRs, those of SL mutants were only half the size compared to the wild-type [41]. Treatment with racGR24 accelerated the CR growth in wild-type and rescued the phenotype of the SL biosynthesis mutant *d10*, whereas no effect was observed in the SL signalling mutant *d14*. Although the root meristem of the mutants *d10* and *d14* is shorter than the one in wild-type, the length of the root cells in the mature zone of the roots is similar. This indicates that SLs affects CR elongation by stimulating cell divisions [41].

The role of SLs in adventitious root (AR) formation is also far from clear (Table 1). At maturity, rice SL mutants were found to have more ARs compared to the wild-type. This obscures the fact that during the first weeks of development the same SL mutants contain fewer ARs then the wild-type, and that when tillers are taken into account, mature mutant plants display fewer ARs per tiller than wild-type plants [45]. It has therefore been postulated that SLs stimulate AR formation. Indeed, treatment with racGR24 increased the number of ARs in the SL biosynthesis mutant *d10*, but not in the SL signalling mutant *d3* [45]. In *A. thaliana* and pea, however, SL mutants had fewer ARs, suggesting a negative role of SLs on LR formation [44]. Treatment of these plants with racGR24 suppressed AR formation in both wild-type and SL biosynthesis mutants in a dose dependent manner but had no effect on SL signalling mutants [44].

In many aspects of root development, the role of SLs remains unclear and inconsistent data for different species have been published (Table 1). It has to be stressed that the availability of nutrients, like phosphorus or nitrogen, modulates the function of SLs in shaping root architecture (see Chapter 3). To clarify the role of SLs in root development it is necessary to investigate SL mutants and related wild-type under controlled conditions, before more complex systems, such as treatment with other hormones are considered.

## 3. Role of SLs in Regulating Root Architecture under Stress Conditions

SLs are involved in plant response to nutrient stress, since elevated SL production and exudations were observed in different species under phosphorus (P) and nitrogen (N) starvation conditions [9]. Plants adapt to low N and P concentrations by changing shoot and root architecture: the development of above-ground parts is stopped whereas root area increases to enhance the uptake nutrients. In *A. thaliana* the inhibition of PR growth is accompanied by an increase in LR density and length [46]. In other species, like rice, an elongation of both SRs and LRs was observed [47]. Whereas in *A. thaliana* grown under optimal growth conditions SLs reduce LR abundancy, the opposite was observed under P starvation where treatment with racGR24 promote outgrowth of LR primordia [36]. A similar positive effect of SLs on LR development under nutrient deficiency was also observed in rice. Studies confirmed that under P and N starvation the production of SLs is enhanced in wild-type plants, which was correlated with elevated expression of the SL biosynthesis genes *D10*, *D17* and *D27*. At the same time expression of SL signalling genes *D3* and *D14* was decreased, probably due to negative feedback regulation [43]. Concerning the remodelling of root architecture, rice SL mutants *d3*, *d10* and *d27* are less sensitive to both N and P starvation conditions than wild-type plants. In the presence of racGR24, however, the sensitivity of SL biosynthesis mutants to N and P deficiency became similar to that observed in wild-type plants, whereas SL signalling mutants showed no such increase in sensitivity [43]. In the presence of the SL biosynthesis inhibitor abamine [48] SR growth in wild-type plants was not affected by P and N starvation, while P-starvation was found to stimulate growth of CRs in wild-type plants, but not in SL mutants [41].

These data prove that the modification of root architecture in response to nutrient stress is SL dependent. Furthermore, SLs appear to play a somewhat different role under nutrient deficiency than under optimal growth conditions (Table 2). For example, SLs inhibited the number of LRs in *A. thaliana* under optimal conditions, but an opposite effect was observed under P starvation [36].

Sanz et al. realized that under P and N deficiency, SLs act together with nitric oxide (NO) in regulating root elongation [49]. Furthermore, rice plants accumulate NO in the root tips under N and P starvation, which is correlated with an increased meristem activity and elongation of SRs [50]. Under the same conditions the SL mutants *d3* and *d10* showed a similar increase in NO in root tip meristems but no root elongation [50]. NO induces the degradation of D53, the repressor of the SL signalling pathway, in wild-type plants but not in the *d14* mutant. This shows that elongation of rice SRs in response to N and P starvation is induced by NO, which in turn depends on degradation of the SL repressor [50]. It is not known yet if a similar mechanism plays a role in aspects of plant development that are modulated by SLs and NO, such as LR and AR development, or plant responses to abiotic stress or pathogen attack [49].

On the other hand increased biosynthesis of SLs, in response to P and N deficiency, is correlated with increased exudation of SLs to the soil [9]. Analyses of the level of SLs in the roots of *Sorghum bicolor* L. under P and N deficiency revealed that the production of 5-deoxystrigol, one of the major SLs identified in this species, was almost 30 times higher during the response to nutrient stress conditions than in the control plants [51]. Similar results were obtained for other legume and non-legume species, such as *Calendula officinalis* L., *Triticum aestivum* L., *Solanum lycopersicum* L., rice, *Trifolium pretense* or *Pisum sativum* L. (reviewed by [9]). It was proposed that the reason of increased production and exudation of SLs under nutrient deficiency is the promotion of symbioses with arbuscular mycorrhizal fungi. This interaction with fungal partner enhanced the root capacity of soil exploration through the fungal mycelium and providing an additional source of macro- and micronutrients for plant partner [52]. Whereas in case of legumes, plants that are using cooperation with N-fixing rhizobial bacteria during N uptake, increased exudation of SLs is related with the stimulation with symbiotic bacteria [6]. It was proved that SLs are involved in promotion of interactions between roots and bacteria, and SLs biosynthesis mutant were characterized as defected in symbiosis with N-fixing bacteria, but on the other hand it was proved that N does not regulate nodulation through SLs [53]. Finally, we have to keep in mind that increased production of SLs inhibits shoot branching, what is a part of the plant response to starvation. It was proved that SL mutants are unable to regulate shoot branching in response to P starvation, what indicated that SL production/perception are required for the suppression of branching under a P deficiency [54,55].

Presented results clearly shown that SLs. are involved in different aspect of plant adaptation to survive under nutrient deficiency conditions. They do not only regulate the root architecture, but also support different strategies to enhance uptake of nutrients and survive the time when nutrient availability is limited.

## 4. How SLs Influence on Root Development

SLs regulate root system architecture via interactions with other hormones, such as IAA and cytokinins (CKs). In *A. thaliana* roots racGR24 treatment causes a decrease and/or altered distribution of the auxin efflux carriers PIN-FORMED1 (PIN1), PIN3, and PIN7 in PRs [42]. RacGR24 also reduced the concentration of PIN3 in LR primordia suggesting that SLs influence the IAA export needed for LR formation [36]. A similar mechanism exists in axillary buds formation where SLs promote the endocytosis of PIN1 which inhibits IAA export [56]. Treatment with racGR24 enhanced the level of IAA in axillary buds which stopped a further outgrowth [38].

A detailed analysis of PIN1 localization after racGR24 treatment failed to confirm the previously described effect [56]. However, racGR24 was found to promote the polar localization of PIN2 in the root epidermis of wild-type plants, but not in SL signalling mutants [57]. Further analysis revealed that a mutation in *PIN7* reduced the root sensitivity to racGR24, and additionally mutant *pin1* is more sensitive to SLs and forms fewer LRs in the presence of racGR24 [58]. Although the cross talk between SLs and IAA may differ between roots and shoot, its undisputed influence on localization and activity of PIN proteins in roots, identify SLs as a potentially main player in the regulation of IAA transport [58].

Since both, SLs and CKs, play a negative role in LR elongation, a cross talk between these two classes of hormones has been postulated. Analysis of CK signalling mutants indicated that mutations in the CK receptor—ARABIDOPSIS HISTIDINE KINASE3 (AHK3)—resulted in insensitivity to racGR24 treatment. Plants with mutations in the CK receptors AHK2 and AHK4, still respond to racGR24 with a reduction in LR density [58]. Analogous to AHK3 which regulates the meristem size by a transcriptional control of IAA induced genes [59], SLs might thus be involved in LR primordia formation.

The fourth class of hormones involved in regulating root development is ethylene. In *A. thaliana* it acts as a negative regulator of AR formation, similar to SLs. While *A. thaliana* SL mutants are sensitive to treatment with the ethylene precursor 1-aminocyclopropane-1-carboxylic acid, mutants in ethylene biosynthesis and signalling are sensitive to racGR24 treatment [60]. When both hormones were applied the response was stronger in comparison to the single treatments, an indication that both phytohormones act independently in the repression of AR development [60].

Metabolome analysis in wild-type and *max2* in the presence or absence of racGR24 revealed a group of metabolites whose production or accumulation is SLs dependent [61]. The majority of these metabolites were flavonols, previously identified as regulators of root development [62] and additionally it was proved that function of flavonols depends on both IAA [63] and abscisic acid (ABA) [64].

Treatment with GR24^5DS^ and GR24^ent-5DS^ recognized by the SL receptor D14 and KAR receptor KAI2, respectively increased, in both cases, the production of flavonols. In the single *d14* and *kai2* mutants flavonols were still present [61], suggesting the existence of a cross-talk between SLs and KARs in roots. Further an analysis of double *kai2* and *d14* mutants may shed new light on the action of both hormones on plant growth and development.

## 5. Concluding Remarks

SLs are best known for their role in the regulation of root system architecture. A detailed analysis of the literature brought to light several inconsistencies regarding the putative function of SLs (Table 1). The simplest explanation is that SLs may modulate their role in root development in response to environmental conditions, in particular the availability of N and/or P. In plant adaptation to nutrient deficiency, they promote LR growth, however with opposite function under optimal growth conditions. As it becomes clear that plant growth conditions modulate SL functions, a precise experimental set-up will be crucial. It is also conceivable that the tap root system of dicots responds differently to SLs than the fibrous root system of monocots. It is therefore essential that phenotypic analyses of SL mutants are performed under controlled conditions. To identify SL-dependent root features studies should be carried out using different growth systems as hydroponics or soil. A final cause of concern is the frequent use of *max2* mutants which apart for SL signalling, are also involved in KAR signalling. A better alternative would be the application of the specific SL signalling mutant *dwarf* (*d14*) which is identified in multiple species, including *A. thaliana* and rice. Finally, for SL treatment experiments only the GR24^5DS^ or pure naturally occurring SLs should be employed. Such studies should be supplemented by detailed investigations of the molecular cross talk between SLs and other main players in root development: IAA or CKs.

## Figures and Tables

**Table 1:** Role of strigolactones (SLs) in root development under optimal growth conditions.

Investigated Species	Tools	Role of SLs	Reference
Primary root (PR)/seminal root (SR) length			
*Solanum lycopersicon* L.	racGR24	Did not influence root length, reversed the suppressive effect of IAA	[42]
*Arabidopsis thaliana* L.	SL biosynthesis (*max3-11*, *max4-1*) and signalling (*max2-1*), racGR24	No effect under optimal conditions, promotion of PR length under carbohydrate starvation	[36]
*Oryza sativa* L.	SL biosynthesis (*d10*, *d17*, *d27*) and signalling (*d3*, *d14*) mutants, racGR24	Lack of effect on SR length	[41]
*Oryza sativa* L.	racGR24	Promotion of SR elongation	[43]
*Hordeum vulgare* L.	SL insensitive mutant (*hvd14.d*), racGR24	Promotion of SR growth	[38]
*Lotus japonicus* L.	RNAi transgenic line for *LjCCD7*	Inhibition of PR	[39]
Lateral root (LR) density			
*Arabidopsis thaliana* L.	SL biosynthesis (*max3-11*, *max4-1*) and signalling (*max2-1*) mutants, racGR24	Inhibition of LR density	[35]
*Arabidopsis thaliana* L.	SL biosynthesis (*max3-11*, *max4-1*) and signalling (*max2-1*) mutants, racGR24	Inhibition of LR density	[36]
*Arabidopsis thaliana* L.	SL signalling (*max2*) mutant, racGR24	Inhibition of LR initials outgrowth	[37]
*Oryza sativa* L.	SL biosynthesis (*d10*) and signalling (*d14*) mutants	No effect in LR density	[41]
*Oryza sativa* L.	racGR24	Inhibition of LR density	[43]
*Hordeum vulgare*	SL insensitive mutant (*hvd14.d*), racGR24	Inhibition of LR density	[38]
*Lotus japonicus* L.	RNAi transgenic line for *LjCCD7*	Inhibition of LR density	[39]
*Medicago truncatula* Gaertn.	racGR24	Inhibition of LR density	[40]
Crown root (CR) length			
*Oryza sativa* L.	SL biosynthesis (*d10*) and signalling (*d14*) mutants	Promotion of CR growth	[41]
Adventitious root (AR) formation			
*Arabidopsis thaliana* L.	SL biosynthesis (*max1*, *max3*, *max4*) and signalling (*max2*), racGR24	Negative effect of AR formation	[44]
*Pisum sativum* L.	SL biosynthesis (*rms1*, *rms5*) and signalling (*rms4*), racGR24	Negative effect of AR formation	[44]
*Oryza sativa* L.	SL biosynthesis (*d10*, *d17*, *d27*) and signalling (*d3*, *d14*) mutants, racGR24	Promotion of AR formation	[45]

**Table 2:** Role of SLs in root response to nutrient stresses.

Applied Stress	Investigated Species	Obtained Results	Reference
P-deficiency (20 µM)	*Arabidopsis thaliana* L.	Increased number of LRs in wild-type under stress conditions, whereas SL mutants contained more LR primordia with arrested outgrowth.	[36]
N-deficiency (0.02 mM)	*Oryza sativa* L.	Under stress promotion of SR elongation was significantly lower in SL mutants in comparison to wild-type; treatment with racGR24 increased SR elongation in SL biosynthesis mutants to the values observed in wild-type, which was not the case in SL signalling mutant.	[43]
P-deficiency (2 µM)			
N-deficiency (0.02 mM)	*Oryza sativa* L.	Treatment with SL biosynthesis inhibitor stopped elongation of SRs in wild-type plants under stress conditions.	[5]
P-deficiency (2 µM)			
Phosphate-removed ½ MS	*Oryza sativa* L.	Elongation of CRs in wild-type under P-deficiency, not observed in SL mutants.	[41]
